# Investigation of injury severity level in truck-related crashes at school zones using mixed generalized ordered models

**DOI:** 10.1371/journal.pone.0318725

**Published:** 2025-02-06

**Authors:** Junhan Cho, Sungjun Lee, Juneyoung Park

**Affiliations:** 1 Samsung Traffic Safety Research Institute, Samsung Fire & Marine Insurance Bldg, Seoul, Republic of Korea; 2 Department of Transportation and Logistics Engineering, Hanyang University ERICA, Ansan, Republic of Korea; 3 Department of Transportation and Logistics Engineering, Smart City Engineering, Hanyang University ERICA, Ansan, Republic of Korea; University of Valencia, SPAIN

## Abstract

The number of truck registrations is steadily increasing in Korea. The proportion of truck deaths compared to the total number of traffic crashes was 23.9%, which is significantly higher than that of other vehicles. In the field of traffic safety, the Children’s Safety Measures Policy by government aims to enhance the safety of children’s commuting routes by expanding school zones. Nonetheless, truck crashes continue to occur in school zones. Therefore, this study analyzed the factors that affect the severity of truck traffic crashes in school zones in order to contribute to safety improvements. In the study, a distinction is made between various levels of severity to determine the factors that contribute to each level. The generalized ordered models were applied to investigate injury severity levels. Moreover, in order to account for heterogeneity issue, the mixed-effects models with random parameters were used for the analysis. These models were constructed using data collected from school zones over a period of recent ten years. The results showed that crashes occurred at night and on the weekend, as well as human factors such as the age of the victim and the gender of the offender, the types of vehicles involved, and the road type, have been identified as important factors contributing to crash severity. By considering the factors that contribute to truck crashes in school zones, it is anticipated that road safety can be enhanced.

## 1. Introduction

### 1.1 Overview

Trucks, designed with cargo areas for freight transportation, are critical for logistics but also contribute significantly to social costs, including traffic congestion, road damage, and severe crashes. Truck crashes, involving large freight vehicles, frequently result in higher rates of severe injuries and fatalities compared to other vehicle types. In 2019, trucks accounted for 12.5% of all traffic crashes but were responsible for 23.9% of traffic crash fatalities, highlighting their disproportionate impact (Korean National Police Agency, 2019). While trucks are involved in fewer overall crashes compared to passenger cars (65.9%) and motorcycles (9.1%), their fatality rate per crash is significantly higher, with trucks contributing to 23.9% of fatalities, compared to 46.9% for passenger cars and 14.9% for motorcycles. This data underscores the elevated risks associated with truck crashes.

The number of registered trucks has steadily increased from 2007 to 2020, intensifying concerns about their impact on traffic safety. To mitigate risks, the 2018 children’s safety measures aimed to reduce fatalities involving children by expanding school zones and enhancing walking environments. School zones, typically within a 300-meter radius of school entrances, were designed to improve safety through speed limits, parking restrictions, and pedestrian protection measures. The 2021 Road Traffic Act further prohibited parking and stopping in these zones, except under specific circumstances such as compliance with police orders.

Despite these policies, truck crashes in school zones remain a significant concern. For example, Incheon City designated 26 new "Cargo Restricted Areas," covering 46% of its school zones, to address risks from large cargo trucks frequently used in construction sites. However, these measures are insufficient to address the complex transportation dynamics in school zones, where large vehicles pose unique dangers to pedestrians, especially children.

Crash severity analysis is a widely used method to identify factors contributing to crashes. Independent variables, such as environmental conditions and driver characteristics, are analyzed to determine their influence on crash severity, which serves as the dependent variable. While severity analyses in broader contexts are well-documented, few studies have specifically examined truck crashes in school zones, leaving a critical research gap.

This study offers novel insights by examining truck crashes in school zones, a critical yet infrequently studied aspect of traffic safety. By focusing on these child-centered environments, this research highlights unique risks associated with truck operations and contributes to filling an important gap in the existing literature. Furthermore, the application of mixed-effects ordered probit and logit models provides a robust methodological framework, allowing for a deeper understanding of unobserved heterogeneity in crash observations.

To address this gap, the present study analyzes risk factors associated with truck crashes using existing literature on truck crashes, crashes involving children, and crash severity. Distinctions are made between various levels of severity to identify factors contributing to each level. Crash data are examined based on crash type, cause, and driver involvement, providing a comprehensive analysis of the factors impacting crash severity in school zones.

### 1.2 Literature review

#### 1.2.1. Truck crash

Zheng et al. [1] investigated factors influencing the severity of truck crashes using data from North Dakota and Colorado between 2010 and 2016. Their goal was to understand the impact of various factors, including company and driver characteristics, vehicle types, and collision details. Gradient boosting was selected for its capability to handle heterogeneous risk factors and complex relationships within the data. The analysis revealed associations between the severity of truck crashes and trucking company attributes, safety inspection values, and driver age.

Zhu & Srinivasan [2] examined truck crashes in 2006 by analyzing data from the Large Truck Crash Causation Study using an ordered probit model. They found that the most severe crashes occurred when a truck and a passenger car collided head-on at an intersection. Choi et al. [3] utilized highway crash data to examine the impact of weather and traffic conditions on the severity of truck crashes. They discovered that in abnormal weather conditions, the severity of truck crashes escalated when there was a high coefficient of speed variation, particularly in downhill sections, bridges, and tunnels. Han et al. [4], focusing on Seoul city, highlighted the higher fatality rates associated with truck crashes and noted the role of protective gear use and increased driving experience in influencing crash outcomes. Dong et al. [5] estimated the effects of traffic, driver, geometry, and environmental characteristics to identify factors that influence truck-related collisions. They identified risk factors using a multinomial logistic model that was based on four collision severity categories. Yuan et al. [6] utilized a 5-year dataset from the Fatal Analysis Reporting System to examine the impact of truck driver-related characteristics on fatal crashes. Azimi et al. [7] employed a random parameter ordered probit model with interaction effects to analyze 10 years of truck rollover crash data. Their findings highlighted significant effects of adverse weather, remote areas, curved road sections, and vehicle weight on crash severity.

These studies collectively examined the severity of truck crashes by evaluating factors such as driver characteristics, collision types, and traffic conditions, providing a robust foundation for understanding the diverse influences on truck crash outcomes.

#### 1.2.2. Children involved crashes

Ko and Park [8] investigated the effects of neighborhood environmental characteristics on pedestrian-vehicle collisions within school zones, utilizing pedestrian-vehicle crash data. Through Geographic Information System (GIS) and spatial econometric analysis, they identified the relationship between the neighborhood environment surrounding school zones and pedestrian traffic crashes. The analysis showed that both road factors and public transportation factors negatively impact pedestrian traffic crashes in school zones. Kim and Park [9] evaluated data from four years of traffic crashes involving children in Incheon and identified the factors that influenced the severity of these crashes using an ordered probit model. They found that the season, signal violation, victim vehicle type, weather, and road surface were significant factors. Jeong [10] analyzed how children’s walking behavior affected the severity of pedestrian traffic crashes and found that the "victim’s behavior type," "crash type," and "gender" were significant factors. Environmental factors, geometric structure factors, school-related factors, and factors related to rotary intersections were considered. The results of all those previous studies show that factors such as the presence of school zones, vehicle types, traffic conditions, and types of crashes are related to the severity of crashes involving children.

#### 1.2.3. Severity prediction

Park [11] examined the severity of pedestrian crashes at four-point intersections using an ordered probit model, categorizing crashes into property damage-only, minor, severe, and fatal crashes. The analysis identified age, time of day, vehicle type, and speed limits as significant contributors to crash severity. Ahn et al. [12] analyzed nationwide data from the Traffic Crash Management System, categorizing crash severity into property damage-only, severe, and fatal crashes. They found that individuals aged 40–50 significantly contributed to crash severity due to drunk driving, alongside road conditions and planar alignment factors. Lee et al. [13] aim to analyze the severity level of injuries among drivers and school-age passengers, as well as identify contributing factors. The study specifically focuses on the impact of driver characteristics on the severity of injuries sustained by both the driver and child passengers. Iranitalab & Khattak [14] evaluated the effectiveness of statistical and machine learning methods in predicting traffic collision severity. Using data from two-vehicle collisions, methods such as Multinomial logit, Nearest neighbor classification, Support Vector Machines, and Random Forests were applied. The analysis showed that statistical models demonstrated superior performance. Ma et al. [15] used an ordered logit model to analyze hazardous material road traffic crashes in China, identifying unsafe driving behaviors and violations as major risk factors. Xie et al. [16] employed a random parameter ordered probit model to study collision severity on two-lane rural roads, accounting for unobserved heterogeneity. Their findings emphasized the significant role of illegal driving behaviors and vehicle types. Garrido et al. [17] used an ordered probit model to analyze injury severity among car occupants, noting that occupants of light vehicles on two-way roads experienced more severe injuries compared to those in heavy vehicles on one-way roads. Yan et al. [18] utilized a binary logit model to analyze car-bicycle collisions using four years of crash data from Beijing. Eboli L et al. [19] used a binary logit model to investigate the effects of road-related factors, environmental factors, driver-related factors, and crash-related factors on different types of road crashes. Aiash & Robuste [20] used a binary probit model to determine the relationship between crash severity and classification factors based on the occurrence of fatalities in the crashes. According to their analysis, the most severe crashes occurred during weekends and at night. Taamneh & Taamneh [21] classified the collisions in six years of Abu Dhabi crash data into four categories and compared the performance of a random forest and ordered probit model in predicting the severity of road crashes. These factors include the age and gender of both the driver and pedestrian, the use of seatbelts, traffic conditions, the time of day, and the type of crash. Most studies used ordered probit models, but these were occasionally supplemented with other models, such as binary logit models. Mannering [22] investigated temporal instability in highway crash data, emphasizing its potential impact on model inferences and predictive capabilities. Kim et al. [23] developed a Mixed logit model to analyze driver injury severity in single-vehicle crashes in California. They used the mixed logit model to account for individual heterogeneity based on age and gender. This model allows for the capture of heterogeneity through random parameters, and it enables explanatory variables to influence the means of these random parameters. The findings indicated that older drivers (aged 65 and above) are more likely to sustain fatal injuries in single-vehicle crashes. However, heterogeneity was observed, indicating varying outcomes among drivers within the elderly population. Okafor et al. [24] investigated the factors contributing to the severity of single-vehicle right run-off-road and left run-off-road crashes. To address the heterogeneity of the observations, they employed a random parameter ordered logit model. The analysis revealed that male drivers, drunk driving, and dry road conditions were significant factors in the severity of these crashes. As discussed, through research on severity prediction, we have observed the application of various statistical models. These studies include the ordered probit, ordered logit, random parameter ordered probit, and random parameter ordered logit models, exploring how each model has been utilized in predicting crash severity. The ordered probit model is effective for classifying different severity levels by maintaining the ordinal structure of data through threshold setting. However, its fixed parameters limit its ability to account for heterogeneity among observations. The ordered logit model, which uses the logit function, is commonly applied in binary classification and severity prediction but shares the limitation of fixed parameters.

In contrast, random parameter models introduce flexibility by incorporating random parameters, enabling the reflection of individual heterogeneity. The random parameter ordered probit model extends the ordered probit model, improving precision in modeling observation-level variability, though it increases estimation and interpretation complexity. Similarly, the random parameter ordered logit model utilizes logit transformation while accounting for variability among observations, making it particularly suited for analyzing diverse crash scenarios in varied traffic environments.

This study evaluates the strengths and limitations of these models to identify the most suitable approach for analyzing crash severity. Unlike traditional studies that predominantly focus on highways, this research uniquely examines the severity of truck crashes within school zones nationwide, addressing a critical and underexplored area of traffic safety.

## 2. Materials and methods

### 2.1. Methods

#### 2.1.1. Ordered probit model

The basic theory of an ordered probit model is as follows. If general ordinal data have a data type in which y goes up to 0, 1, 2, 3, …, the ordered probit model can be expressed as follows [25–29]:

$$\begin{array}{l} {\text{y}}^{*}=\beta {\text{X}}_{\text{i}}+\epsilon_{\text{i}}\text{,}\epsilon_{\text{i}}\sim \text{N}\left[0,1\right]\\ \text{y}=0\;\;\text{if}\;{\text{y}}^{*}<\text{0}\\ \text{y}=1\;\text{if}\;\text{0}\;\leq {\text{y}}^{*}<\mu_{1}\\ \text{y}={\text{y}}_{\text{i}}\;\text{if}\;\mu_{\text{y}-1}\leq {\text{y}}^{*} \end{array}$$ (1)

Here, y is a non-measurable latent utility that can be expressed as a measurable utility (*βX*_*i*_) and a non-measurable utility (*ε*_*i*_). *μ* is called the threshold value, and it is estimated together with the estimation coefficient of each explanatory variable to calculate the selection probability for the choice alternative. Additionally, y* is a latent continuous variable that represents the unobserved propensity underlying the ordinal outcome. The selection probability for each alternative (according to the order) can be expressed as Eq (2).


$$\begin{array}{l} \text{Prob}[\text{y}=\text{0}]=\phi (-\beta \text{x})\\ \text{Prob}[\text{y}=\text{1}]=\phi (\mu_{1}-\beta \text{x})-\phi (-\beta \text{x})\\ \text{Prob}[\text{y}={\text{y}}_{\text{i}}]=\text{1}-\phi (\mu_{{\text{y}}_{\text{i}}-1}-\beta \text{x}) \end{array}$$ (2)

The marginal effect that expresses the influence of each explanatory variable on the severity of the crash can be expressed by Eq (3).


$$\begin{array}{l} \frac{\partial \;\text{Prob}\left[\text{y}=0\right]}{\partial \;\text{x}}=-\phi \left(\beta \text{x}\right)\beta \\ \frac{\partial \;\text{Prob}\left[\text{y}=1\right]}{\partial \;\text{x}}=\left[\phi \left(-\beta \text{x}\right)\beta -\phi \left(-\beta \text{x}\right)\right]\beta \\ \frac{\partial \;\text{Prob}\left[\text{y}=2\right]}{\partial \;\text{x}}=-\phi \left(\mu -\beta \text{x}\right)\beta \end{array}$$ (3)

This can be expressed by applying a partial differential to each explanatory variable. For example, in the case of a dummy variable (X = 0 or 1), the marginal effect indicating the influence of the explanatory variable on crash severity expresses the difference in the selection probability between a case in which the variable is 1 and a case in which the variable is 0 when the other explanatory variables are fixed. Therefore, the sum of the marginal effects for each explanatory variable is zero. In the finally derived model, the model verification method uses likelihood ratio to indicate the explanatory power of the model and *χ*^2^ (chi-squared) to verify the suitability of the model.

#### 2.1.2. Ordered logit model

An ordered logit model is an extension of logistic regression analysis that is used when the dependent variables are categorical and the categories (or levels) have a significant order of three or more. The ordinal variable Y is a function of another variable that is continuous, unmeasured, and has multiple critical points. The value of the observed variable depends on whether a certain threshold is exceeded, as shown in the following equations [30].


$$\begin{array}{l} {\text{Y}}_{\text{i}}=1\;\text{if}\;{\text{Y}}_{\text{i}}^{*}<{\text{k}}_{1}\\ {\text{Y}}_{\text{i}}=\text{j}\;\text{if}\;{\text{k}}_{\text{i}}\;\leq {\text{Y}}_{\text{i}}^{*}<{\text{k}}_{\text{i}-1}\\ {\text{Y}}_{\text{i}}=\text{M}\;\text{if}\;{\text{Y}}_{\text{i}}^{*}\geq {\text{k}}_{\text{M}-1} \end{array}$$ (4)

The continuous latent variables are:

$${\text{Y}}_{\text{i}}^{*}=\overset{\text{K}}{\underset{\text{k}=1}{\sum }}\beta_{\text{k}}{\text{X}}_{\text{ki}}+\epsilon_{\text{i}}$$ (5)

There is a random disturbance term (*ε*_*i*_) that follows a normal distribution. The error term reflects the fact that variables might not be fully measured and that some related variables might not be present in the equation. The parameters are estimated using the maximum likelihood method, and the suitability of a generally ordered logit model is verified by the Nagelkerke R2. The statistical effects of the variables are based on the p-value in the Wald test [19].

#### 2.1.3. Mixed effects ordered probit model

Theoretically, ordered probit models do not allow parameters to vary across observations. To address the issue of heterogeneity, mixed-effects models (also known as random-parameters models) were developed to accommodate the variation of certain parameters across crash observations. The mixed-effects ordered probit models consider the unobserved heterogeneity across individual observations by adding a random term to the estimated coefficient of explanatory variables. In this paper, we applied this approach to explore the key variables that significantly affect severity. Formally, the standard ordered probit model can be represented as follows [16]:

$$y_{i}^{*}=\sum_{k=1}^{K}\beta_{k}X_{ik}+\varepsilon_{i}=\beta X_{i}+\varepsilon_{i}=Z_{i}+\varepsilon_{i}$$ (6)

Where, $y_{i}^{*}$ is the latent oucome variable; *β*_*k*_ is the estimated coefficient of the kth independent variable; *X*_*ik*_ is the kth independent variable of the ith sample; *ε*_*i*_ is the random error term, and it is assumed to be a normal distribution with a mean equal to 0 and a variance equal to 1; *β* is the vector of the estimated coefficient; the estimated value *Z*_*i*_ for ith sample can be expressed as:

$$Z_{i}=\sum_{k=1}^{K}\beta_{k}X_{ik}.$$ (7)

However, as mentioned in previous studies, the estimated outcome from standard ordered probit model may be biased due to neglecting the heterogeneity in individual observations [31–33]. To obtain the unobserved heterogeneity of each independent variable, a randomly distributed term *σ*_*i*_, was added to the estimated coefficient *β*.

$$\beta_{i}=\beta +\sigma_{i}$$ (8)

Where, *β*_*i*_ is the random coefficient vector. Based on the thresholds of *μ*_0_, *μ*_1_ and *μ*_2_, the dependent variable *y*_*i*_ can be determined as follows:

$$y_{i}=\left\{\begin{array}{c} 0\;\left(Minor\;injury\right),\;if\;y_{i}^{*}<\mu_{0}\\ 1\left(severe\;injury\right),\;if\;\mu_{0}\leq y_{i}^{*}<\mu_{1}\\ 2\left(fatal\;severe\right),\;if\;y_{i}^{*}\geq \mu_{1} \end{array}\right.$$ (9)

#### 2.1.4. Mixed effects ordered logit model

The mixed-effects ordered logit model is a suitable method for exploring the contributing factors of crash severity, as the dependent variable has an ordered nature. The model consists of fixed parameters (same effects across observations) and random parameters that exhibit significant variation across observations. The mixed-effects ordered logit model can be defined as follows [34, 35]:

$$Y_{ij}^{*}=X_{i}\beta_{ij}+\varepsilon_{ij}$$ (10)

Where, $Y_{ij}^{*}$ is the latent continuous variable for observation *i* and severity *j*; *X*_*i*_ is the set of dependent variables; *β*_*ij*_ is the vector of estimable parameters; *ε*_*ij*_ is the independent and identically distributed error term.

To determine the source of variations among observations, interaction variables can be introduced to the model. The random parameters could be defined as follows:

$$\beta_{ij}=\beta +\tau I_{i}+S\varphi_{ij}$$ (11)

Where, *U* is the injury severity outcome; *β*_*ij*_ is the vector of estimable parameters; *β* is the fixed part of coefficient means, which is the same for all observations; *τ* is the interaction variables; *I*_*i*_ is the interaction coefficients; *S* is the matrix of standard deviations; *φ*_*ij*_ is the random derives from the standard normal distribution.


$$\text{U}=\left\{\begin{array}{c} 0\;\left(Minor\;injury\right),\;if\;Y_{ij}^{*}<\mu_{0}\\ 1\left(severe\;injury\right),\;if\;\mu_{0}\leq Y_{ij}^{*}<\mu_{1}\\ 2\left(fatal\;severe\right),\;if\;Y_{ij}^{*}\geq \mu_{1} \end{array}\right.$$

It is presumed that the coefficient of the random parameters follows a normal distribution among observations. The random parameters reveal the existence of heterogeneity, but they cannot identify the factors that caused the variations among observations.

### 2.2. Data preparation

This study utilized national traffic crash data from the Traffic Crash Analysis System (TAAS), maintained by the Korea Road Traffic Authority [36]. TAAS collects, integrates, and analyzes traffic crash data from police and insurance companies to provide comprehensive information for developing effective traffic safety policies.

Our analysis focused on truck crashes occurring in school zones across Korea between 2011 and 2020. School zones are designated areas within a 300-meter radius of a school’s primary entrance, established to enhance the safety of children. The dataset classified crash outcomes into three categories: fatal injuries, severe injuries, and minor injuries.

We collected detailed information on the time and location of each crash, its cause and type, driver characteristics (offending and victim drivers), vehicle attributes, and road and environmental conditions. A total of 504 truck crashes were analyzed to identify factors influencing crash severity. Table 1 summarizes the descriptive statistics of the factors considered in the analysis.

**Table 1:** Descriptive statistics for the explanatory variables.

Type	Variable	Number of observations	Percentage
Severity	Fatal injury	15	2.98%
	Severe injury	233	46.23%
	Minor injury	256	50.79%
Offender gender	Man	488	95.13%
	Woman	16	4.87%
Offender age	21–30 years old	43	8.53%
	31–40 years old	82	16.27%
	41–50 years old	148	29.37%
	51–60 years old	142	28.17%
	61 years old and over	89	17.66%
Victim gender	Man	339	67.64%
	Woman	165	32.36%
Victim age	10–20 years old	481	95.51%
	21 years old and over	23	4.49%
Crash type	Car-to-pedestrian	435	86.16%
	Car-to-car	69	13.84%
Collision type	Crossing	274	53.41%
	Roadway collision	31	6.04%
	Side collision	37	7.21%
	Sidewalk collision	21	4.09%
	Other	141	29.24%
Vehicle type of victim	Pedestrian	435	86.16%
	Bicycle	52	10.53%
	Other	17	3.31%
Time of the day	Day	448	88.89%
	Night	56	11.11%
Day type	Weekday	435	86.35%
	Weekend	69	13.65%
Road type	Other Single road	220	43.65%
	Intersection	160	31.75%
	crosswalk	88	17.46%
	Other	36	7.14%
Roadway grade	Flat	436	86.35%
	Uphill	26	5.46%
	Downhill	42	8.19%
Weather condition	Sunny	468	91.62%
	Cloudy	17	3.51%
	Rainy	19	4.09%

## 3. Results

### 3.1. Estimated models

This study analyzed the severity of truck crashes in school zones nationwide from 2011 to 2020 using ordered probit, ordered logit, and multilevel mixed-effects models. To identify the factors contributing to varying severity levels and to develop effective countermeasures, crash severity was categorized into three levels: minor, severe, and fatal. This classification reflects the potential seriousness of truck crashes, particularly those involving heavy loads.

Table 2 presents the results of the ordered probit and ordered logit models. Factors with statistically significant effects on crash severity were identified at a 95% confidence level. In the model results, positive estimates indicate an increase in crash severity, while negative estimates suggest a decrease. For example, variables such as nighttime, Sundays, the victim being a pedestrian, and the victim being in a van showed positive estimates, indicating a higher likelihood of severe outcomes. In contrast, negative estimates were observed for variables such as the offender being male and an increase in the victim’s age, suggesting a reduced likelihood of severe crash outcomes.

**Table 2:** Results of ordered probit and logit model on injury severity.

Ordered Probit			Ordered Logit		
Variable	Estimate	P-value	Variable	Estimate	P-value
Night	0.39	0.03	Night	0.71	0.02
Sunday	0.60	0.04	Sunday	1.06	0.05
Victim age	-0.05	0.01	Victim age	-0.11	0.02
Victim_pedestrian	0.79	0.00	Victim_pedestrian	1.26	0.00
Victim _van	2.25	0.01	Victim _van	4.71	0.01
Gender of offender	-0.80	0.01	Gender of offender	-1.44	0.01
cut1	-0.57	-	cut1	-1.50	-
cut2	1.41	-	cut2	2.14	-
AIC	778.24		AIC	776.58	

These findings provide important insights into the factors influencing crash severity and can inform the development of targeted interventions and safety policies.

In the mixed-effects ordered probit and logit models, variables listed in Table 3 were derived to address heterogeneity caused by unobserved effects. This study utilizes mixed ordered models to identify factors significantly impacting injury severity in truck-related crashes. Factors with statistically significant effects were estimated at an 85% confidence level. Consistent with the ordered probit and logit model results, variables such as nighttime, Sundays, the victim being a pedestrian, and the victim being in a van showed positive estimates, indicating an increase in crash severity. Conversely, negative estimates were observed for cases where the offender was male, and when crashes occurred at crosswalks, intersections, on other single roads, or involved an increase in the victim’s age, suggesting a decrease in crash severity. In car crashes, pedestrians older than 60 had a higher risk of fatality and severe injuries compared to younger individuals [37]. Crashes involving pedestrians over the age of 45 have also been shown to have a high likelihood of severe injuries [38]. In other words, previous research has shown that the probability of severe or fatal crashes increases as pedestrians age [39]. In contrast, our results show that the severity of crashes decreased as the age of the victim increased. However, as indicated by the descriptive statistics, over 90% of the victims in our analysis were between the ages of 10 and 20. This young age group was particularly susceptible to traffic crashes, highlighting their vulnerability. Our results indicate that various social systems are needed, such as providing traffic safety education about school zones to both truck drivers and children.

**Table 3:** Results of mixed effects ordered probit and logit model on injury severity.

Mixed Effects Ordered Probit			Mixed Effects Ordered Logit		
Variable	Estimate	P-value	Variable	Estimate	P-value
Night	0.64	0.02	Night	1.12	0.03
Sunday	0.94	0.05	Sunday	1.73	0.05
Gender of offender	-1.39	0.01	Gender of offender	-2.54	0.01
Victim_ van	3.76	0.02	Victim_ van	6.96	0.02
Crosswalk	-0.87	0.07	Crosswalk	-1.57	0.07
Intersection	-0.72	0.07	Intersection	-1.35	0.06
Victim age	-0.09	0.02	Victim age	-0.17	0.02
Victim_pedestrian	0.92	0.00	Victim_pedestrian	1.60	0.00
Other single road	-0.56	0.15	Other single road	-1.02	0.15
Constant	2.24	0.02	Constant	4.24	0.01
Cut 1	3.23	0.00	Cut 1	5.98	0.00
AIC	775.60		AIC	776.00	

Large-scale inter-vehicle crashes, such as those involving buses and SUVs, increase the likelihood of injuries. Collisions between trucks and buses, in particular, have a high potential for fatalities [40]. According to previous studies, crashes involving vans and trucks are associated with a higher proportion of severe injuries due to their greater mass and aggressivity [41]. In this research, crash severity increased when the victim vehicle was a van. Clearly, in a collision between a truck and a van, the severity of the crash will increase as the weight of the vehicles increases. We also found that crash severity increased when the victim was a pedestrian. This was expected because in such crashes, the vehicle impact is delivered directly to the pedestrian’s body. Among the characteristics of crash types, it has been shown that pedestrians increase the severity of traffic crashes [42]. Zahabi et al. [43] estimated the factors that affect pedestrian injury severity and found that road design, traffic environment, and other factors were relevant. They also found that these factors worsened at night. In this study, crash severity also increased at night, presumably because darkness reduced the truck driver’s field of vision. Therefore, appropriate visual signals are needed in areas designated for the protection of children. Our study found that the severity of crashes decreased when the offending driver was male. According to previous studies, female drivers are more likely to sustain severe injuries in specific crash scenarios due to differences in driving behaviors. For instance, Russo et al. [44] observed that female drivers exhibited greater injury severity during daylight and under dry road conditions. Similarly, Yan et al. [45] identified a higher likelihood of severe injuries among female drivers in crashes influenced by temporal and environmental factors. As indicated by the descriptive statistics, this is likely attributed to the fact that over 90% of the truck drivers involved in crashes are male. Crashes that occurred on Sundays had greater severity than those on other days of the week, presumably because truck drivers drove carelessly inside school zones on Sundays. According to previous studies, the proportion of fatal crashes is higher for weekend crashes [46]. To address this issue, it is necessary to implement traffic safety education programs focusing on weekend driving. These programs should target both truck drivers and children, as accidents occurring on Sundays, a key part of the weekend, were found to have higher severity.

In an analysis of driver injuries, Abdel-Aty [47] demonstrated that curved roads, dark conditions, vehicle speed, and intersections are variables that correlate with the severity of crashes. In this study, crash severity decreased at intersections, presumably because the truck drivers reduced their speed as they approached the intersection. Factor such as crossing the street at crosswalks decreases injury severity [48]. The study also found that being in a crosswalk reduced the severity of crashes. The lower probability of severe injuries may be attributed to the presence of crosswalks and related pedestrian crossing signs. These signs alert drivers to the potential presence of pedestrians, prompting them to drive more cautiously. Other single road means do not include tunnels, underpasses, or overpasses. Crashes occurring near tunnels, underpasses, and overpasses had a higher probability of severe injuries [49]. In this research, crash severity decreased when the road type was other single road.

### 3.2. Marginal effects

An accurate interpretation of the model is achieved through a marginal effects analysis, which quantifies the impact of independent variables on crash severity. The marginal effect estimates the change in crash severity resulting from a one-unit increase in an independent variable (or a change from 0 to 1 for dummy variables) using partial differentiation. A positive coefficient indicates an increase in crash severity, while a negative coefficient signifies a decrease.

**Fig 1:**
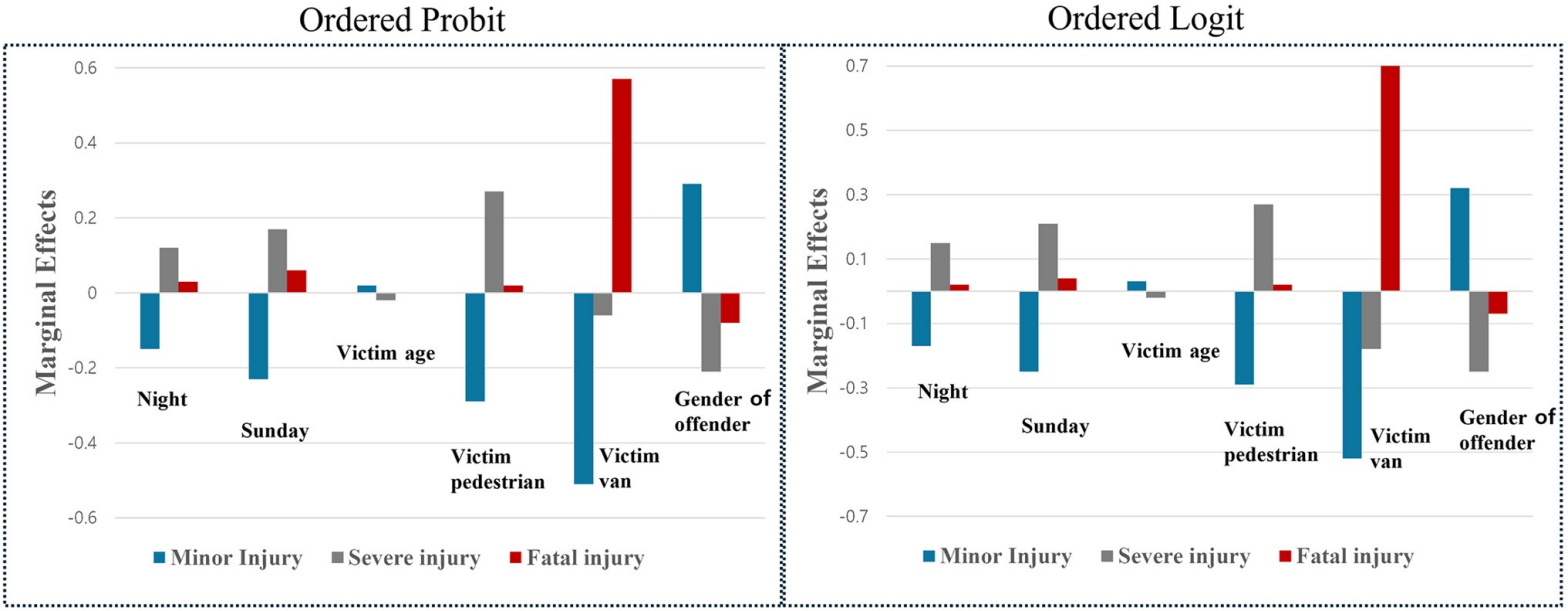
Marginal effects results for ordered probit and ordered logit models.

Table 4 summarizes the marginal effects of the ordered probit model, and Fig 1 visualizes these effects alongside those of the ordered logit model. The analysis revealed that crash severity increased at night, with severe injuries and fatal crashes rising by 12% and 3%, respectively, compared to daytime crashes, likely due to reduced visibility. Crashes occurring on Sundays also showed higher severity, with probabilities of severe injuries and fatalities increasing by 17% and 6%, respectively, compared to weekdays, possibly reflecting more careless driving in school zones on weekends.

**Table 4:** Marginal effects of the ordered probit and ordered logit model.

Ordered Probit				Ordered Logit			
Variable	Minor Injury	Severe injury	Fatal injury	Variable	Minor injury	Severe injury	Fatal injury
Night	-0.15	0.12	0.03	Night	-0.17	0.15	0.02
Sunday	-0.23	0.17	0.06	Sunday	-0.25	0.21	0.04
Victim age	0.02	-0.02	0.00	Victim age	0.03	-0.02	0.00
Victim_pedestrian	-0.29	0.27	0.02	Victim_pedestrian	-0.29	0.27	0.02
Victim_ van	-0.51	-0.06	0.57	Victim_ van	-0.52	-0.18	0.70
Gender of offender	0.29	-0.21	-0.08	Gender of offender	0.32	-0.25	-0.07

When the victim was a pedestrian, the severity was markedly higher, with probabilities of severe injuries and fatalities increasing by 27% and 2%, respectively, compared to vehicle-to-vehicle crashes. Crashes involving vans as victim vehicles showed mixed outcomes: the probability of minor and severe injuries decreased by 51% and 6%, respectively, while the probability of fatalities increased by 57% compared to passenger cars. This heightened severity in van crashes may be attributed to the larger number of passengers and the substantial impact between heavy vehicles, particularly school vehicles.

Among human factors, crash severity decreased by 2% with an increase in the victim’s age, reflecting that most victims in school zones are children, whose cognitive and coping abilities improve with age. Additionally, crashes involving male offenders had lower severity, with probabilities of severe injuries and fatalities decreasing by 21% and 8%, respectively, compared to crashes involving female offenders.

Table 4 also shows the results of the marginal effect analysis of the ordered logit model, and they are similar to those from the ordered probit model. At night, the likelihood of severe injury and death increased by 15% and 2%, respectively, compared to daytime crashes. On Sunday, the probability of severe injury and death increased by 21% and 4%, respectively, compared to weekday crashes. When the victim is a pedestrian, the probability of severe injury and death increases by 27% and 2% respectively, compared to vehicle-to-vehicle crashes. If the victim’s vehicle was a van, the probability of a fatal crash increased by 70% compared to victims in cars. Among the human factors, the probability of severe injury decreased by 2% as the age of the victim increased. If the offender was male, the probability of severe injury and fatalities decreased by 25% and 7%, respectively, compared to crashes in which the offender was female.

**Fig 2:**
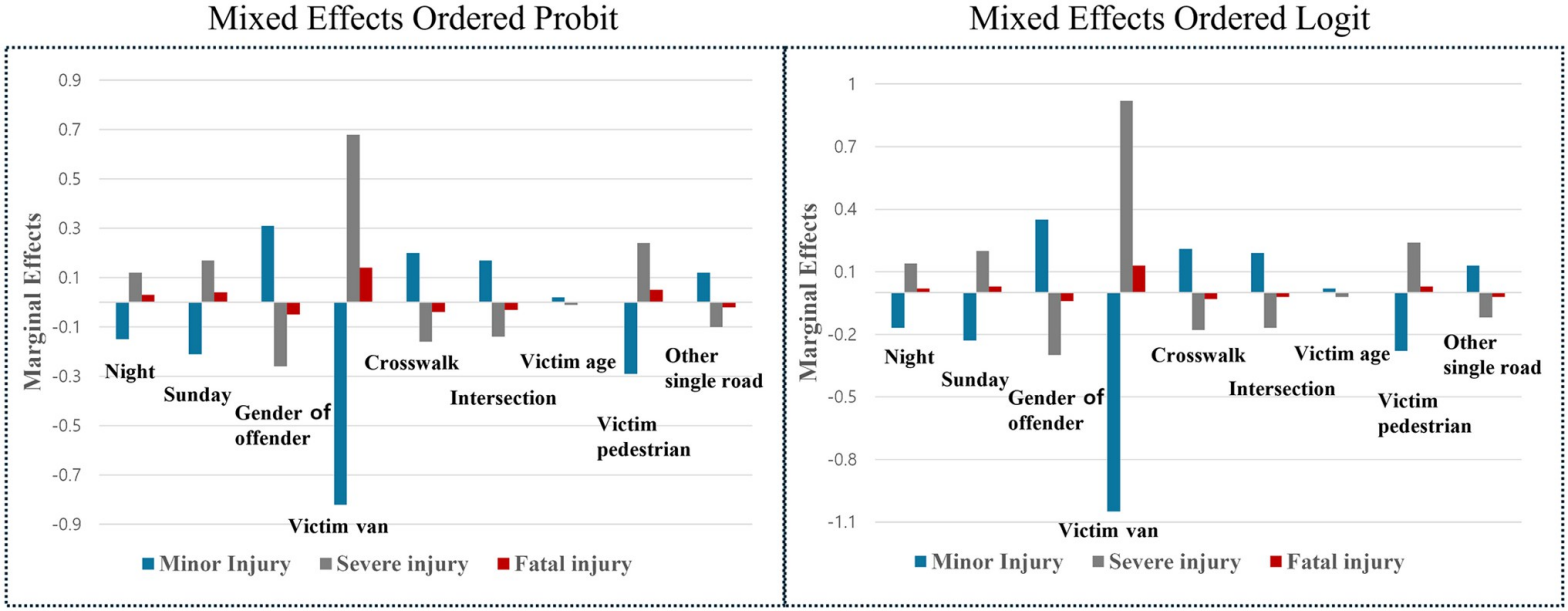
Marginal effects results for mixed effects ordered probit and ordered logit models.

Table 5 presents the marginal effects analysis results for the mixed-effects probit model, with Fig 2 visualizing the marginal effects for both the mixed-effects ordered probit and logit models. The marginal effects of variables such as nighttime, Sunday, increasing victim age, victim being a pedestrian, victim being in a van, and offender gender showed patterns consistent with those observed in the ordered models.

**Table 5:** Marginal effects of the mixed effects ordered probit and ordered logit model.

Mixed Effects Ordered Probit				Mixed Effects Ordered Logit			
Variable	Minor Injury	Severe injury	Fatal injury	Variable	Minor injury	Severe injury	Fatal injury
Night	-0.15	0.12	0.03	Night	-0.17	0.14	0.02
Sunday	-0.21	0.17	0.04	Sunday	-0.23	0.20	0.03
Gender of offender	0.31	-0.26	-0.05	Gender of offender	0.35	-0.30	-0.04
Victim_ van	-0.82	0.68	0.14	Victim_ van	-1.05	0.92	0.13
Crosswalk	0.20	-0.16	-0.04	Crosswalk	0.21	-0.18	-0.03
Intersection	0.17	-0.14	-0.03	Intersection	0.19	-0.17	-0.02
Victim age	0.02	-0.01	0.00	Victim age	0.02	-0.02	0.00
Victim_pedestrian	-0.29	0.24	0.05	Victim_pedestrian	-0.28	0.24	0.03
Other single road	0.12	-0.10	-0.02	Other single road	0.13	-0.12	-0.02

The probability of severe injuries and fatalities decreased by 16% and 4%, respectively, when the road type was a crosswalk. Similarly, intersections were associated with a 14% and 3% reduction in the likelihood of severe injuries and fatalities. On "other single roads," the probabilities of severe and fatal crashes decreased by 10% and 2%, respectively.

Table 5 also shows the results of the marginal effect analysis of the mixed-effects logit model. The two models showed similar results. The probability of severe injury and fatality decreased by 18% and 3%, respectively, when the road type was a crosswalk. The probability of severe injury and fatality decreased by 17% and 2% respectively when the road type was an intersection. In addition, the probability of severe and fatal crashes decreased by 12%, and 2% for other single road.

### 3.3. Model comparison

This study compared crash severity using four models and evaluated their goodness of fit using the Akaike Information Criterion (AIC), a widely used metric for model comparison proposed by Akaike [50]. A threshold of 2.5 was applied to the AIC difference to identify the preferred model, based on previous research [51].

In the ordered probit and logit models, key variables included crashes occurring at night or on Sundays, victim age, whether the victim was a pedestrian or in a van, and the gender of the offender. The mixed-effects ordered probit and logit models also considered additional variables, such as the presence of crosswalks, intersections, and other single road segments.

**Fig 3:**
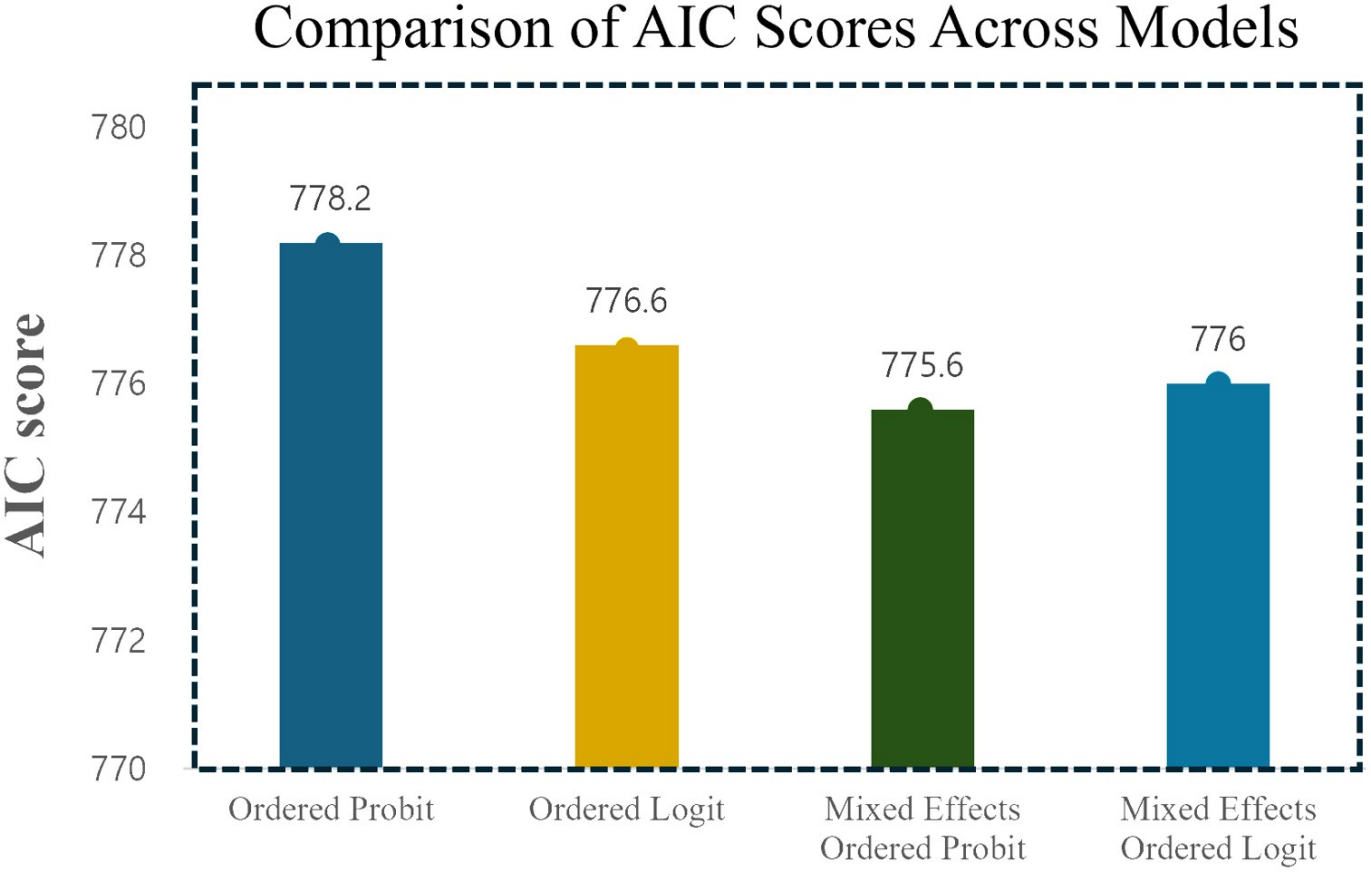
Comparison of AIC scores across models.

Fig 3 visualizes the AIC values for the four models. The ordered probit and logit models yielded AIC values of 778.24 and 776.58, respectively. The mixed-effects ordered probit and logit models showed lower AIC values of 775.6 and 776, respectively, indicating improved explanatory power. Among these, the mixed-effects ordered probit model had the lowest AIC value, demonstrating the best fit to the data.

This comparison highlights the superior explanatory capability of mixed-effects models compared to basic ordered models, emphasizing their suitability for analyzing crash severity in this study.

## 4. Discussion

This study analyzed 504 truck-related crashes in school zones and identified key factors influencing crash severity. This study offers novel insights by examining truck crashes in school zones, a critical yet understudied aspect of traffic safety. The distinctive environmental and behavioral characteristics of school zones necessitate focused attention to address the unique risks posed by truck operations in child-centered environments. By targeting this specific context, the research sheds light on previously unexplored factors contributing to crash severity. Moreover, the methodological framework employed in this study sets it apart from prior research. The use of mixed-effects ordered probit and logit models with random parameters allows for a robust exploration of unobserved heterogeneity across crash observations, enhancing the depth of the analysis.

Nighttime and weekends were associated with higher severity, while crosswalks and intersections reduced severity. Human factors, including the victim’s age and the offender’s gender, significantly impacted the outcomes, with younger victims experiencing more severe injuries. Vehicle type, particularly crashes involving pedestrians and vans, also played a critical role in increasing crash severity.

The increased severity of nighttime crashes can be attributed to reduced visibility, limiting drivers’ ability to detect pedestrians and respond to road conditions. Crashes on Sundays likely reflect reduced vigilance among drivers, potentially due to less traffic enforcement or relaxation of driving standards on weekends. Meanwhile, the presence of crosswalks and intersections likely encouraged reduced vehicle speeds, mitigating crash impacts. The heightened severity in crashes involving vans is linked to the larger number of passengers and the greater mass involved in such collisions, while pedestrian-involved crashes had inherently higher risks due to direct physical impact.

The findings suggest the need for targeted safety interventions in school zones. Enhancing nighttime visibility through improved lighting and reflective signage could mitigate risks during dark hours. Enforcing stricter traffic regulations on weekends, such as deploying additional monitoring systems, could help reduce the severity of Sunday crashes. Road infrastructure improvements, including the addition of crosswalks and intersection signage, would encourage safer driving practices. Education programs for truck drivers and children could also play a pivotal role in reducing crashes, particularly by improving awareness of high-risk behaviors and areas.

The data, sourced from a single country, may restrict the generalizability of findings to other contexts. Future research could explore these factors by incorporating data from multiple regions and investigating the temporal variations in crash severity trends. Further studies could also examine the interactions between environmental, vehicular, and human factors to develop more comprehensive safety measures.

## 5. Conclusions

In this study, we employed ordered probit, ordered logit, mixed-effects ordered probit, and mixed-effects ordered logit models to investigate various factors influencing truck crash severity. These models effectively categorized variables affecting crash severity, including environmental factors, crash characteristics, and human factors. The analysis demonstrated that models incorporating random parameters, namely the random parameter ordered probit and random parameter ordered logit models, yielded lower AIC values compared to standard ordered models, indicating superior explanatory power. This finding underscores the ability of random parameter models to better account for heterogeneity in crash data and capture random effects across diverse observations.

The significance of utilizing random parameter models lies in their capacity to reflect the unique conditions of individual crash cases. Unlike standard models, random parameter models acknowledge that crashes occur under varying circumstances, allowing for more precise analyses tailored to specific contexts.

However, one limitation of this study is the potential for bias arising from the extensive time period covered by the data. Temporal instability in crash trends could influence the results. Future research should address this limitation by analyzing data for each year separately and exploring interactions between years and other variables. Extending the application of random parameter models and further examining heterogeneity across crash cases could yield deeper insights.

By addressing these considerations, future studies can enhance the understanding of factors influencing truck crashes in school zones, ultimately contributing to the development of more effective safety measures and improving road safety outcomes.
