# Correction: Investigation of injury severity level in truck-related crashes at school zones using mixed generalized ordered models

**DOI:** 10.1371/journal.pone.0323286

**Published:** 2025-04-29

**Authors:** Junhan Cho, Sungjun Lee, Juneyoung Park

The following information is missing from the Funding statement: This work was supported by the Korea Institute of Police Technology (KIPoT), funded by the Korea National Police Agency (KNPA), grant RS-2023-00260576, for the development of traffic accident reproduction software for autonomous vehicle accident investigation and analysis.
